# Human CD79b^+^ neutrophils in the blood are associated with early-stage melanoma

**DOI:** 10.3389/fimmu.2023.1224045

**Published:** 2023-10-31

**Authors:** Melissa A. Meyer, Huy Q. Dinh, Ahmad Alimadadi, Daniel J. Araujo, Nandini Chatterjee, Norma A. Gutierrez, Yanfang Peipei Zhu, Emma L. Hunter, Shu Liang, Gregory Seumois, William B. Kiosses, Sergio D. Catz, Pandurangan Vijayanand, Christian Ottensmeier, Catherine C. Hedrick

**Affiliations:** ^1^ Center for Cancer Immunotherapy, La Jolla Institute for Immunology, La Jolla, CA, United States; ^2^ McArdle Laboratory for Cancer Research, Department of Oncology, University of Wisconsin-Madison, Madison, WI, United States; ^3^ Department of Pediatrics, School of Medicine, University of California, San Diego, San Diego, CA, United States; ^4^ School of Cancer Sciences, University of Southampton Faculty of Medicine, Southampton, United Kingdom; ^5^ Microscopy and Histology Core Facility, La Jolla Institute for Immunology, La Jolla, CA, United States; ^6^ Department of Molecular Medicine, The Scripps Research Institute, La Jolla, CA, United States; ^7^ Institute of Translational Medicine, Department of Molecular & Clinical Cancer Medicine, University of Liverpool, Liverpool, United Kingdom; ^8^ Immunology Center of Georgia, Department of Medicine, Medical College of Georgia at Augusta University, Augusta, GA, United States

**Keywords:** neutrophils, melanoma, biomarker, mass cytometry, CD79b

## Abstract

**Purpose:**

Due to their abundance in the blood, low RNA content, and short lifespan, neutrophils have been classically considered to be one homogenous pool. However, recent work has found that mature neutrophils and neutrophil progenitors are composed of unique subsets exhibiting context-dependent functions. In this study, we ask if neutrophil heterogeneity is associated with melanoma incidence and/or disease stage.

**Experimental design:**

Using mass cytometry, we profiled melanoma patient blood for unique cell surface markers among neutrophils. Markers were tested for their predictiveness using flow cytometry data and random forest machine learning.

**Results:**

We identified CD79b^+^ neutrophils (CD3^-^CD56^-^CD19^-^Siglec8^-^CD203c^-^CD86^Lo^CD66b^+^CD79b^+^) that are normally restricted to the bone marrow in healthy humans but appear in the blood of subjects with early-stage melanoma. Further, we found CD79b^+^ neutrophils present in tumors of subjects with head and neck cancer. AI-mediated machine learning analysis of neutrophils from subjects with melanoma confirmed that CD79b expression among peripheral blood neutrophils is highly important in identifying melanoma incidence. We noted that CD79b^+^ neutrophils possessed a neutrophilic appearance but have transcriptional and surface-marker phenotypes reminiscent of B cells. Compared to remaining blood neutrophils, CD79b^+^ neutrophils are primed for NETosis, express higher levels of antigen presentation-related proteins, and have an increased capacity for phagocytosis.

**Conclusion:**

Our work suggests that CD79b^+^ neutrophils are associated with early-stage melanoma.

## 1 Introduction

Melanoma, and many other cancers, lack a simple blood test to detect cancer incidence. The efforts to create a blood-based test have focused on the quantification of microRNAs, cell free DNA (cfDNA), tumor-associated antibodies, and circulating tumor cells (CTCs) (1–6). Yet, no clinical blood-based test is available for early melanoma disease detection.

Human neutrophils are the most abundant immune cell type in the blood, where they operate as the first line of defense against invading pathogens (7). Neutrophils have traditionally been described as a population with little phenotypic flexibility owing to their short lifespans and low RNA content (8). However, contemporary work suggests that human neutrophils are more diverse than previously understood. For example, while specific human neutrophil populations suppress T cell activity (8, 9), others operate as antigen presenters and T cell stimulators (10–13). Various other neutrophil functions, such as cytokine production, phagocytosis, and NETosis, are now known to be dynamic and context-specific (14, 15). Further, neutrophils have a high turnover rate, with an average half-life of less than one day in the blood (16). Many neutrophil progenitor pools are restricted to the bone marrow during steady-state but can be found in the periphery during human disease (9, 17–19). For instance, our laboratory has shown that NePs (9) and eNePs (17), two early neutrophil progenitors, are both elevated in the blood of melanoma subjects. The abundance of neutrophils in the blood, their unappreciated heterogeneity, phenotypic changes in the periphery during disease, and high turnover makes neutrophils poised to act as potent and responsive biomarkers.

In this study, we aimed to define further the heterogeneity of blood neutrophils in human subjects with melanoma. We also sought to identify neutrophil-based markers that could be associated with melanoma incidence and disease stage.

## 2 Materials and methods

### 2.1 Human bone marrow cells

Fresh bone marrow collected from anonymous healthy donors was obtained from AllCells, Inc. (Alameda, CA) and shipped overnight on a cold pack. Heparin was used as an anti-coagulant. Cells were immediately processed upon arrival for flow cytometry, cell sorting, or *in vitro* assays. Sorted cells were used for cytospin, bulk RNA-seq, *in vitro* assays, and cellular imaging. All available donor characteristics have been listed in **Supplementary Table 1**.

### 2.2 Human peripheral blood collection

Biospecimen Repository Core Facility (BRCF) at the University of Kansas Cancer Center provided peripheral blood from untreated melanoma subjects prior to resection. All melanoma cases were cutaneous. Blood was collected into EDTA-coated tubes and shipped overnight on a cold pack. Upon arrival, cells were processed fresh for mass cytometry, flow cytometry, ImageStream, cell sorting, or *in vitro* assays. Peripheral blood from healthy donors was acquired from the La Jolla Institute for Immunology Normal Blood Donor Program in EDTA-coated tubes and processed for mass cytometry, flow cytometry, and cell sorting. Samples were processed on the day of the blood draw if the sample stood alone or placed at 4°C overnight if the sample was used as a control for melanoma patient samples shipped overnight. The second cohort of samples from melanoma subjects and age-matched healthy donors were obtained from Southampton University Hospitals, UK. Melanoma subject blood was collected prior to treatment and resection. All melanoma cases were cutaneous. Blood was collected in EDTA-coated tubes and processed for mass cytometry. Samples were processed fresh the day of the blood draw if a healthy donor and melanoma patient sample matched in age. Or, healthy donor samples were placed at 4°C overnight prior to processing if the sample was used as a control for melanoma patient samples shipped overnight. Summarized subject characteristics for each cohort have been listed in **Tables 1**–**3**. All available subject characteristics have been listed in **Supplementary Table 1**.

**Table 1:** Subject demographic for CyTOF analysis.

	Melanoma
**Number of Subjects**	6
Age	
Median	74
Range	51-78
Sex	
Male	3 (60%)
Female	2 (40%)
Race	
White British	4 (80%)
South African	1 (20%)
Cell Composition, mean (range)	
Cell count per mL	N/A
% neutrophils of total	71.74 (59.4-86.2)
% CD79b^+^ neutrophils of total	1.365 (0.1759-5.307)
% CD79b^+^ neutrophils of neutrophils	1.722 (0.28-6.45)

**Figure 1:**
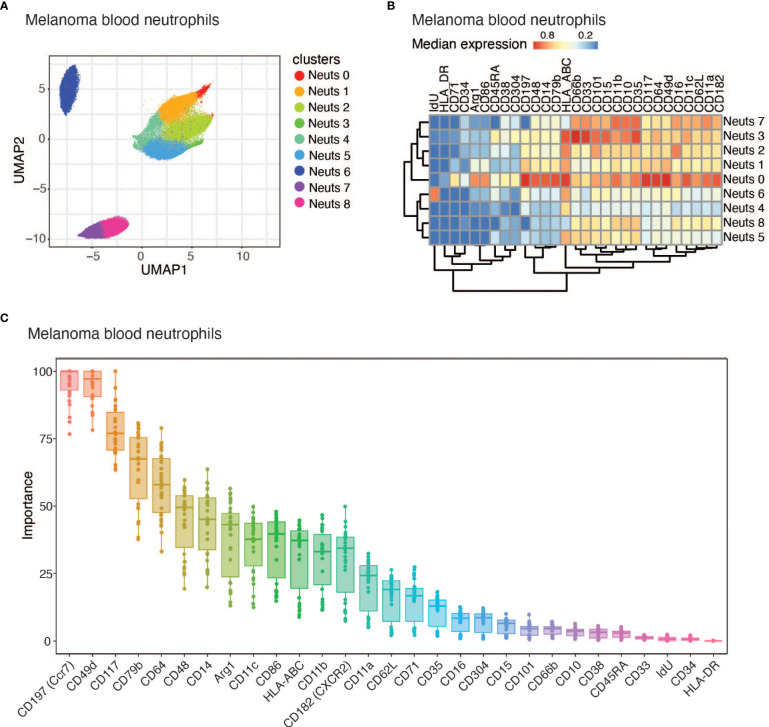
CD79b expression identified among melanoma subject blood neutrophils. **(A)** UMAP projection of melanoma subject blood neutrophil (CD3^-^CD127^-^CD56^-^CD86^Lo^CD19^-^CD66b^+^) CyTOF data displaying all 9 identified neutrophil clusters using PhenoGraph clustering. n=5 biological replicates. **(B)** Heatmap of the median of normalized expression of CyTOF protein markers of nine identified clusters of neutrophils pooled from n=5 samples. **(C)** Importance for each marker in determining whether a cell belongs to Neuts 0 or another cluster using Random Forest machine learning method.

Summarized subject demographics for samples used in CyTOF analysis that are displayed in **Figure 1**. Full available subject characteristics are available in **Supplemental Table 1**.

**Table 2:** Subject demographic for flow cytometry analysis.

	Healthy	Melanoma
**Number of Subjects**	12	41
Age		
Median	33.5	64
Range	26-48	24-87
Sex		
Male	6 (50%)	22 (54%)
Female	6 (50%)	19 (46%)
Race		
White	0 (0%)	41 (100%)
N/A	12 (100%)	0 (0%)
Stage		
1		10 (24%)
2		6 (15%)
3		8 (20%)
4		1 (2%)
N/A		16 (39%)
Cell Composition, mean (range)		
Cell count per mL	N/A	1.9 x 10^7^ (2.4 x 10^6^-7.25 x 10^7^)
% neutrophils of total	53.8 (43.1-62.4)	59.5 (21.8-82)
% CD79b+ neutrophils of total	0.04574 (0.01894-0.08275)	1.112 (0.02776-4.304)
% CD79b+ neutrophils of neutrophils	0.08812 (0.03422-0.1796)	2.215 (0.0395-10.01)

**Figure 2:**
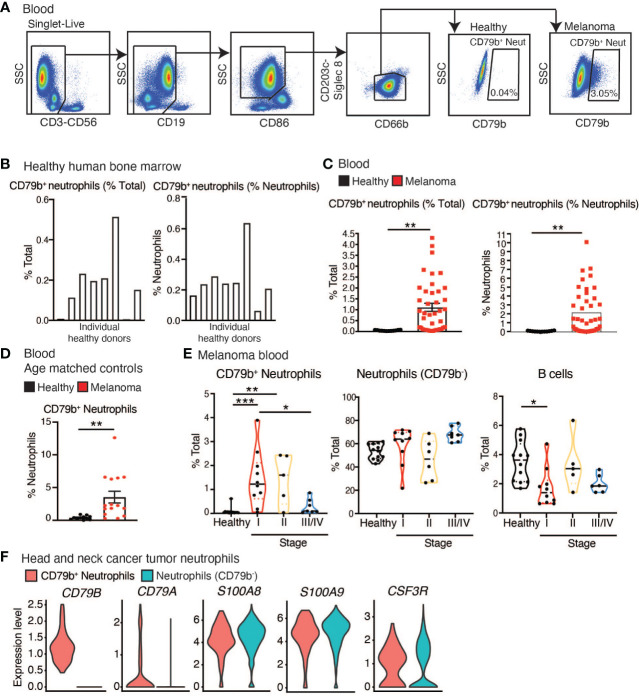
CD79b^+^ neutrophils are enriched in the periphery of melanoma subjects. **(A)** CD79b^+^ neutrophil gating strategy used in human melanoma subject blood. **(B)** Frequency of CD79b^+^ neutrophils (CD3^-^CD56^-^CD19^-^CD203c^-^Siglec8^-^CD86^Lo^CD66b^+^CD79b^+^) in healthy human bone marrow as a percentage of total live cells and as a percentage of neutrophils (CD3^-^CD56^-^CD19^-^CD203c^-^Siglec8^-^CD86^Lo^CD66b^+^), n=8 biological replicates. **(C)** CD79b^+^ neutrophils measured in the melanoma subject blood and healthy control subjects by flow cytometry, n=41 melanoma subjects, n=12 healthy controls. **(D)** Quantification of CD79b^+^ neutrophils in blood of melanoma subjects and healthy age-matched controls by CyTOF as a percentage of neutrophils, n=11 healthy controls, n=13 melanoma subjects. **(E)** Data from **(C)** divided based on subject stage. Healthy n=12, Stage 1 n=10, Stage 2 n=6, Stage III/IV n=9. **(F)** Violin plots show the identification of CD79b^+^ neutrophils (*CD79A/B*+) among head and neck cancer tumoral neutrophils in scRNA-seq data. Tumoral neutrophils were identified by high expression of *CSF3R* (17% out of 8,781 myeloid cells and 1.15% out of 133,180 CD45^+^ cells). CD79b^+^ neutrophils (0.33% of neutrophils) exclusively expressed *CD79A* (FDR-adjusted p-value <1e-42) and *CD79B* (FDR-adjusted p-value 1.1917e-41, Wilcox test) while retaining high expression of neutrophil markers (*S100A8/9* and *CSF3R*, FDR-adjusted p-value 1, Wilcox test).* p<0.05, ** p<0.01, and *** p<0.001 by two-sided, unpaired t-test for single comparisons and by one-way ANOVA with Tukey’s *post hoc* test for multiple comparisons.

Summarized subject demographics for samples used in flow cytometry analysis that are displayed in **Figures 2C–E**. Full available subject characteristics are available in **Supplemental Table 1**.

**Table 3:** Subject demographic for age-matched CyTOF analysis.

	Healthy	Melanoma
**Number of Subjects**	11	13
Age		
Median	69	64
Range	50-81	24-87
Sex		
Male	4 (36%)	5 (38%)
Female	7 (64%)	8 (62%)
Race		
White	10 (90%)	12 (92%)
*British White*	*10 (90%)*	*5 (38%)*
N/A	1 (10%)	1 (8%)
Stage		
1		2 (15%)
2		2 (15%)
3		1 (8%)
4		0 (0%)
N/A		8 (62%)
Cell Composition, mean (range)		
Cell count per mL	N/A	N/A
% neutrophils of total	68.5 (60.7-79.2)	60.33 (21.8-86.2)
% CD79b^+^ neutrophils of total	0.2782 (0-0.6396)	1.286 (0.1749-5.307)
% CD79b^+^ neutrophils of neutrophils	0.3978 (0-0.91)	2.506 (0.28-6.45)

Summarized subject demographics for samples used in CyTOF analysis that are displayed in **Figure 2D**. Full available subject characteristics are available in **Supplemental Table 1**.

### 2.3 Cell sorting

3-5 mL of healthy human bone marrow or human melanoma subject blood were treated with cooled 1x red blood cell lysis buffer (Biolegend) at room temperature (30 mL x 5 minutes, 20 mL x 5 minutes). Cells were resuspended in sterile-filtered FACS buffer [PBS with 1% Human AB Serum (Millipore Sigma) 2mM EDTA (Gibco) 1mg/mL NaN_3_ (Sigma)]. Cells were treated with Live/Dead stain and titrated antibodies in FACS buffer using master mixes at a concentration of 5 million cells per 100μl for 30 minutes at 4°C in the dark. Cells were then washed with FACS buffer and filtered through 70μm strainers. Cells were sorted on BD FACSMelody or BD FACSARIA, received into TriZOL LS Reagent (ThermoFisher Scientific), and frozen immediately at -80°C or received into 10% FBS in PBS and immediately used for cytospin, cellular imaging, or *in vitro* assays. Flavopiridol and recombinant RNAse inhibitors (40U/mL; Takara Bio USA, Inc.) were added to all solutions when sorting for use in bulk RNA sequencing.

### 2.4 Cytospin

Cells were sorted as described above and then cytocentrifuged onto microscope slides using a Cytospin 4 centrifuge (Thermo Shandon). Slides were stained with Hema 3 Manual Staining Systems, and images were collected by light microscopy.

### 2.5 Flow cytometry

Whole human blood and bone marrow cells were processed in cooled 1x red blood cell lysis buffer (Biolegend) at room temperature (30 mL x 5 minutes, 20 mL x 5 minutes) and washed with FACS buffer (PBS with 1% Human AB Serum (Millipore Sigma) 2mM EDTA (Gibco) 1mg/mL NaN_3_ (Sigma)). Cells used in *in vitro* assays were only washed with FACS buffer. All samples were aliquoted at no more than 5 million cells per well and treated with 100μl Live/Dead staining and titrated antibody master mixes for 30 minutes at 4°C in the dark. Cells were washed with FACS buffer and then fixed with 4% paraformaldehyde (PFA) for 10 minutes at 4°C in the dark. All samples were washed again with FACS buffer and filtered through 70μm strainers. For DAPI and Sytox Orange (SO) NETosis assay, cells were stained according to the protocol in Zharkova et al., 2019 (20). In short, cells were fixed using 1.3% PFA and stained with 0.3nM DAPI and 0.1μM SO for 30 minutes. Cells were acquired on an LSR Fortessa (BD Bioscience) and analyzed using FlowJo software (BD). Gating strategies have been indicated in the figures and text. Antibodies used have been listed in **Supplementary Table 4**.

### 2.6 Bulk RNA-sequencing

Cells were sorted into TriZOL LS and frozen at -80°C, as described above. RNA isolation was performed using Direct-zol RNA MiniPrep (Zymo Research) according to the manufacturer’s instructions. RNA concentrations were measured using an Agilent 2200 TapeStation and Agilent High Sensitivity RNA ScreenTape System (Agilent Technologies). Library preparation and sequencing were performed using the Smart-Seq2 protocol (21). RNA-Seq short reads were mapped onto the human genome (hg38) using subread-align from the Subread R package (22). Uniquely mapped reads were annotated with NCBI RefSeq annotation using featureCounts R package (v1.5.3) (23) and used for downstream differential analysis. Multidimensional scaling (MDS) analysis and differential expression testing were performed using linear model analysis (function voom from limma R package; v3.33.7) (24) with scaling normalization factors estimated using edgeR package (25). Multi-test correction using Benjamini and Hochberg method was implemented using the p.adjust function in R (26). Data are available on Gene Expression Omnibus (GEO) under accession code GSE154777.

### 2.7 Mass cytometry

CyTOF staining was performed according to a previously described protocol (27). 1 mL human blood was processed in cooled 1x red blood cell lysis buffer (Biolegend) at room temperature (30 mL x 5 minutes, 20 mL x 5 minutes). Cells were stained with 5μM Cisplatin (Fluidigm) in phosphate-buffered saline (PBS, Corning) to measure viability. Cells were then blocked by resuspending in the staining buffer for 15 minutes at room temperature. Master mixes of titrated antibodies were applied for 1 hour at 4°C. Cells were washed with staining buffer and fixed with 1.6% PFA for 15 minutes at room temperature. Intercalator solution [125nM Cell-ID Intercalator-IR (Fluidigm) in Maxpar Fix and Perm Buffer (Fluidigm)] was applied in equal volume, mixed, and then removed. Cells were resuspended in intercalator solution and incubated overnight at 4°C. Afterward, cells were washed with staining buffer. Cells from the second cohort of subjects (UK Southampton) were then resuspended in 5% DMSO (Sigma) in FBS and slow frozen at -80°C for later washing and acquisition as follows. All other samples were washed with Cell Acquisition Solution (CAS) (Fluidigm) and then resuspended in CAS with 1:10 diluted EQ Four Element Calibration bead (Fluidigm) and filtered through 35μm strainers (Corning). Samples were acquired on a Helios 2 CyTOF Mass Cytometer (Fluidigm) equipped with a Super Sampler (Victorian Airship & Scientific Apparatus). The bead-based Normalizer was used to normalize samples (28).

Data were pre-processed to obtain single, live Lin^-^CD66b^+^ cells. Lineage included CD3, CD127, CD56, CD86, and CD19. These data were further analyzed using R and Bioconductor packages. Protein expression was normalized using arcsinh transformation (cofactor=5). We used Phenograph clustering (29) implemented in the cytofkit package (30) to identify CD79b^+^ neutrophils. Consensus clustering was used to justify the number of clusters from k=2 to k=30, based on relative decreases in the area under the CDF (Cumulative Distribution Function) curve (31). We then merged the clusters based on their consensus clustering to divide CD79b^+^ neutrophils from CD79b^-^ neutrophils. Heatmaps of median protein expression were produced using the pheatmap R package (v0.2).

### 2.8 Random forest machine learning

To evaluate markers among blood neutrophils that distinguish healthy donors from melanoma subjects and cluster Neuts 0 from other cluster, we used Random Forest (RF) machine learning method based on R framework (version 4.1.0) and R packages, such as caret version 6.0-92 (32) and randomForest version 4.7-1.1 (33) were used to calculate variable importance scores. Each dataset contained CyTOF marker expression and two conditions (Healthy/Melanoma or Neuts 0/Others). RF models were trained using the caret package “train” function for thirty iterations with 10000 and 3000 randomly-selected cells in each iteration for each dataset, respectively. Before the training stage, data were scaled and centered. The resampling method was set to repeatedcv, with 10 folds, 10 repeats, and tune_length of 10. The variable importance was estimated and scaled using the “varImp” function from the caret package.

### 2.9 Reanalysis of head and neck cancer neutrophils

scRNA-Seq data was downloaded from NCBI GEO (GSE139324) (34) and reanalyzed in a fashion similar to what was done with the scRNA-Seq above to determine myeloid compartments. Neutrophils were sorted out *in silico* using the expression of *S100A8/9* and *CSF3R*, according to a recent study focused on the landscape of tumor-infiltrating myeloid cells in lung cancer (35). CD79b^+^ neutrophils were then selected out of neutrophils using *CD79A/B* expression.

### 2.10 ImageStream

Human melanoma subject blood was prepared and stained as described in the flow cytometry section above. Before the acquisition, cells were permeabilized using a Permeabilization Buffer (eBioscience) and stained with Hoechst 33342 (Invitrogen) diluted 1:2500 in PBS for 5 minutes at 4°C. Cells were then washed with PBS. Afterward, images were acquired on Amnis ImageStreamX MarkII (Luminex) at Sanford Burnham Prebys Medical Discovery Institute.

### 2.11 Netosis assay

Human melanoma subject blood and healthy human bone marrow were processed in cooled 1x red blood cell lysis buffer (Biolegend) at room temperature (30 mL x 5 minutes, 20 mL x 5 minutes). Cells were washed and resuspended in 0.5% Human AB Serum (Millipore Sigma), 10mM HEPES (Gibco), 2mM L-Glutamine (Gibco), 1x Penicillin-Streptomycin (Gibco) in RPMI-1640 (Gibco) +/- 10μM PMA and aliquoted at 5 million cells/200μl in 96 well round bottom plates. Cells were incubated at 37°C and 5% CO_2_ for 3 hours. Cells were then washed with FACS buffer and stained as described under flow cytometry above.

### 2.12 Confocal imaging

Sorted CD79b^+^ neutrophils were fixed with 4% PFA for 10 minutes at room temperature. Cells were washed with PBS, then blocked and permeabilized with 1% BSA and 0.02% saponin in PBS for 1 hour at room temperature. CD66b-PE (1:400) and CD79b-Alexa647 (1:100) were added, and samples were incubated at 4°C overnight. Cells were washed with PBS. Phalloidin (1:500, Molecular Probes) and Hoechst (1:1000, Sigma) were used to stain in PBS for 1 hour. Cells were washed with PBS and then mounted in Prolong Gold (Molecular Probes). For enhanced-resolution microscopy, single-image z-stacks with an average of 36 slices were acquired on an LSM Airyscan 880 laser-scanning confocal microscope (63x 1.4 NA objective, z-step size 0.17 μm). Post-acquisition, 16-bit images were linearly deconvolved and automatically Airyscan-processed using the dedicated ZEN software (ver3.0) module. Identical acquisition settings were used for all acquired experimental samples, including laser power and detector signal amplification (digital gain). This included controls where baseline intensity thresholds were defined by both cellular autofluorescence and secondary antibody intensity ranges for each experiment. All images were post-processed as maximum intensity projections using the ZEN software and also 3D rendered using Imaris software vers 9.8 (Bitplane Inc) for figure panels.

### 2.13 Antigen uptake studies

Human melanoma subject blood was processed in cooled 1x red blood cell lysis buffer (Biolegend) at room temperature (30 mL x 5 minutes, 20 mL x 5 minutes). Cells were washed and resuspended in 0.5% Human AB Serum (Millipore Sigma), 10mM HEPES (Gibco), 2mM L-Glutamine (Gibco), 1x Penicillin-Streptomycin (Gibco) in RPMI-1640 (Gibco) and aliquoted at 5 million cells per well in a round bottom 96 well plate. 5ul Zymosan particles (abcam) or 5 million B16F10-ZsGreen tumor cells were added to blood cells. B16F10-ZsGreen tumor cells were obtained from Matthew Krummel at the University of California San Francisco. Plates were incubated at 37°C and 5% CO_2_ for 2 hours. Cells were washed with FACS buffer and stained as described under flow cytometry above without fixation.

### 2.14 B cell – neutrophil co-culture

Neutrophils and B cells were sorted from healthy human blood as described in the cell sorting section above. Neutrophils alone, B cells alone, and neutrophils and B cells at a 1:1 ratio were plated in a round bottom 96 well plate in 2.5% autologous serum and 1x Penicillin-Streptomycin (Gibco) in RPMI-1640 (Gibco). Plates were incubated at 37°C and 5% CO_2_ for either 4 or 24 hours. Cells were washed with FACS buffer and stained as described under flow cytometry above.

### 2.15 ROS and superoxide assays

Human melanoma subject blood was processed in cooled 1x red blood cell lysis buffer (Biolegend) at room temperature (30 mL x 5 minutes, 20 mL x 5 minutes). Cells were stimulated and stained using a ROS/Superoxide Detection Assay Kit (abcam) according to the manufacturer’s instructions. Cells were further stained as described in the flow cytometry section above.

### 2.16 Statistics

GraphPad Prism and R were used to conduct statistical tests and plot the data. Data are plotted as mean +/- SEM. Unpaired, two sided t-tests were used to compare two groups as indicated in the figure legends. One-way ANOVA or RM one-way ANOVA with the Geisser-Greenhouse correction with Tukey’s *post hoc* tests were used to compare more than two groups, as indicated in the figure legends. p values of less than 0.05 were considered significant.

### 2.17 Human Study approval

Peripheral blood from healthy donors was collected at La Jolla Institute after written and informed consent was obtained under the guidelines of the Institutional Review Board of La Jolla Institute for Immunology and in accordance with US Department of Health and Human Services Policy for the Protection of Human Research Subjects (VD-057-0217). Peripheral blood from melanoma subjects was collected at Southampton University Hospitals, UK, after written, informed consent was obtained from all participants under the guidelines from the Southampton and Southwest Hampshire Research Ethics Committee and the Institutional Review Board of the La Jolla Institute for Immunology, and in accordance with US Department of Health and Human Services Policy for protection of Human Research Subjects.

## 3 Results

### 3.1 CD79b expression identified in blood neutrophils from melanoma subjects

To better understand the heterogeneity amongst peripheral neutrophils during cancer, we profiled whole blood from subjects with melanoma by mass cytometry (CyTOF) using a panel of 40 protein markers. Five patient blood samples were collected from the University of Southampton (**Table 1** and **Supplementary Table 1**). All melanoma subjects have cutaneous melanoma and were treatment naïve, and blood samples were collected prior to resection. We clustered neutrophils (CD3^-^CD127^-^CD56^-^CD86^Lo^CD19^-^CD66b^+^) using Phenograph clustering (29) implemented in the cytofkit package (30) and found 9 subclusters (**Figures 1A, B**). Neutrophil cluster 0 (Neuts 0) stood out because of its unique cell surface expression pattern. First, it expresses CD79b, a marker typically found on B cells that composes part of the B cell receptor. Second, Neuts 0 also expressed several proteins involved in antigen presentation and trafficking, including HLA-DR, HLA-ABC, CD86, and CD197 (**Figure 1B**). Interestingly, the Neuts 0 pool also expressed higher levels of proteins usually found on neutrophil progenitors, such as CD49d, CD117, CD48, CD38, and CD71, compared to other peripheral neutrophils (17, 18). Furthermore, these cells expressed higher Arginase 1 (ARG1) levels, a marker often observed on tumor-associated, immature myeloid cells (36). Finally, subcluster Neuts 0 expressed CD45RA, a potential marker of neutrophil activation (37).

When comparing Neuts 0 to other melanoma blood neutrophils in total, we noticed that this cluster differentially expressed several proteins (**Figure 1B**). Using the Random Forest machine learning method, we identified the importance of each marker in distinguishing Neuts 0 from other blood neutrophil clusters in melanoma subjects. The most distinguishing markers included CD49d and CD117, reported markers of neutrophil progenitors previously found to be enriched in the blood during disease (**Figure 1C**) (17, 38, 39). CD197, another key marker, was previously found to be important for neutrophil trafficking to lymph nodes and was expressed among a subset of tumor-associated neutrophils (**Figure 1C**) (11, 13, 40). CD79b, a B cell marker not previously reported as biologically relevant on neutrophils, also emerged among the top candidates for distinguishing cluster Neuts 0 (**Figure 1C**). As CD79b was previously unexplored in neutrophils, we evaluated CD79b expression in neutrophils from independent cohorts of melanoma subjects using flow cytometry. We further explored co-expression of CD79b and some of the other highly expressed markers in cluster Neuts 0 by flow cytometry. We found CD117 and CD14 were not co-expressed significantly with CD79b, suggesting additional heterogeneity existed within cluster Neuts 0 (**Supplemental Figure 1A**). We did find CD197 was co-expressed with CD79b among neutrophils. On average, 60% of CD79b^+^ neutrophils expressed CD197 in melanoma patient blood (**Supplemental Figure 1A**).

### 3.2 CD79b^+^ neutrophils are enriched in the periphery of melanoma subjects

CD79b^+^ neutrophils were quantified in blood from 41 human melanoma subjects and in the bone marrow of 5, and in the blood of 12 healthy human donors, using flow cytometry (**Table 2** and **Supplementary Table 1**). All melanoma subjects had cutaneous melanoma, were treatment naïve, and blood was collected prior to resection. Bone marrow was sourced from AllCells; healthy human blood was sourced from La Jolla Institute for Immunology’s Normal Blood Donor program; and all melanoma patient blood was sourced from Biospecimen Repository Core Facility (BRCF) at the University of Kansas Cancer Center. Neutrophils were defined as CD3^-^CD56^-^CD19^-^Siglec8^-^CD203c^-^CD86^Lo^CD66b^+^ cells, and the number of CD79b^+^ cells among neutrophils was measured (**Figure 2A** and **Supplemental Figure 1B**). CD79b^+^ neutrophils were present in the bone marrow but absent from the blood of healthy human donors but were elevated in the blood of melanoma subjects (**Figures 2A–C**). In contrast, neutrophils were not elevated in the blood of our melanoma cohort (**Supplemental Figure 1C**). Because CD79b is commonly expressed in B cells, we also measured changes in B cell frequencies within the blood of these subjects. Again, we found that B cells did not change in frequency within melanoma subjects relative to healthy controls (**Supplemental Figure 1C**). CD79b^+^ neutrophils were elevated in melanoma subjects not only as a percentage of total blood cells but also as a percentage of total neutrophils, suggesting that peripheral neutrophil heterogeneity has changed in cancer (**Figure 2C**). Interestingly, CD79b^+^ neutrophils made up less of the neutrophils compartment as neutrophils expanded, suggesting other neutrophil subpopulations dominate as the neutrophil compartment expands (**Supplemental Figure 1D**).

As the healthy controls in this cohort possessed a lower mean age than the melanoma subjects, we asked if the presence of peripheral CD79b^+^ neutrophils correlated with age. Using flow cytometry, we were unable to show any correlation between the frequency of peripheral CD79b^+^ neutrophil frequency and subject age (**Supplemental Figure 1E**). We validated these findings in a second cohort of melanoma subjects and age-matched, healthy controls by using manual gating of our CyTOF data. 11 healthy donors and 13 melanoma subjects were analyzed (**Table 3** and **Supplementary Table 1**). Again, the melanoma subjects have cutaneous melanoma, are treatment naïve, and prior to resection. Healthy human blood was sourced from La Jolla Institute for Immunology’s Normal Blood Donor program or the University of Southampton, and melanoma patient blood was either sourced from Biospecimen Repository Core Facility (BRCF) at the University of Kansas Cancer Center or the University of Southampton. We again found that CD79b^+^ neutrophils were elevated in the peripheral blood of melanoma subjects (**Figure 2D**). When comparing the cohort of melanoma patients listed in **Table 2** to the cohort described in **Table 3**, no statistical differences in CD79b^+^ neutrophils was found (% CD79b^+^ neutrophils of total, p=0.4258, % CD79b^+^ neutrophils of neutrophils, p=0.359 by Mann Whitney test).

We then returned to the larger, initial flow cytometry cohort to analyze CD79b^+^ neutrophils by disease stage (**Table 2** and **Supplementary Table 1**). Patients were segregated into the following disease stages: stage 1 which included 10 subjects, stage 2 which included 6 subjects, and stage 3/4 which included 9 subjects. Importantly, we found that CD79b^+^ neutrophils were enriched in the blood of melanoma subjects with stage I and II tumors but were diminished in subjects with stage III/IV tumors (**Figure 2E**). These results were contrary to levels observed for other remaining neutrophils (CD79b^-^) and B cells in the blood (**Figure 2E**). These data indicate that CD79b expression on peripheral blood neutrophils represents a previously unidentified change in melanoma patients and is specifically associated with early-stage melanoma.

We next asked if we could identify the presence of CD79b^+^ neutrophils in other human tumors. We analyzed a scRNA-seq dataset derived from human head and neck tumor samples (34) and identified 8,781 infiltrated myeloid cells from 133,180 CD45^+^ cells. We identified infiltrating neutrophils in head and neck tumors by selecting the myeloid cells with the highest expression of CSF3R (17% of myeloid cells, 1.15% of CD45^+^ cells) *in silico*, similar to what was used to define neutrophils in CD45^+^ cells in a recent myeloid lung cancer atlas (35). Among those neutrophils, 0.3% expressed *CD79A/B* while retaining high expression of the neutrophil marker *S100A8/9* (**Figure 2F**). Similarly, we analyzed peripheral blood cell scRNA-seq data from the same study using a similar method. We found neutrophils made up 5.85% of peripheral blood mononuclear cells, and 2% of neutrophils expressed *CD79B* (**Supplemental Figure 1F**). These data show that CD79b^+^ neutrophils can be found in the periphery and tumor of subjects with head and neck cancer.

**Figure 3:**
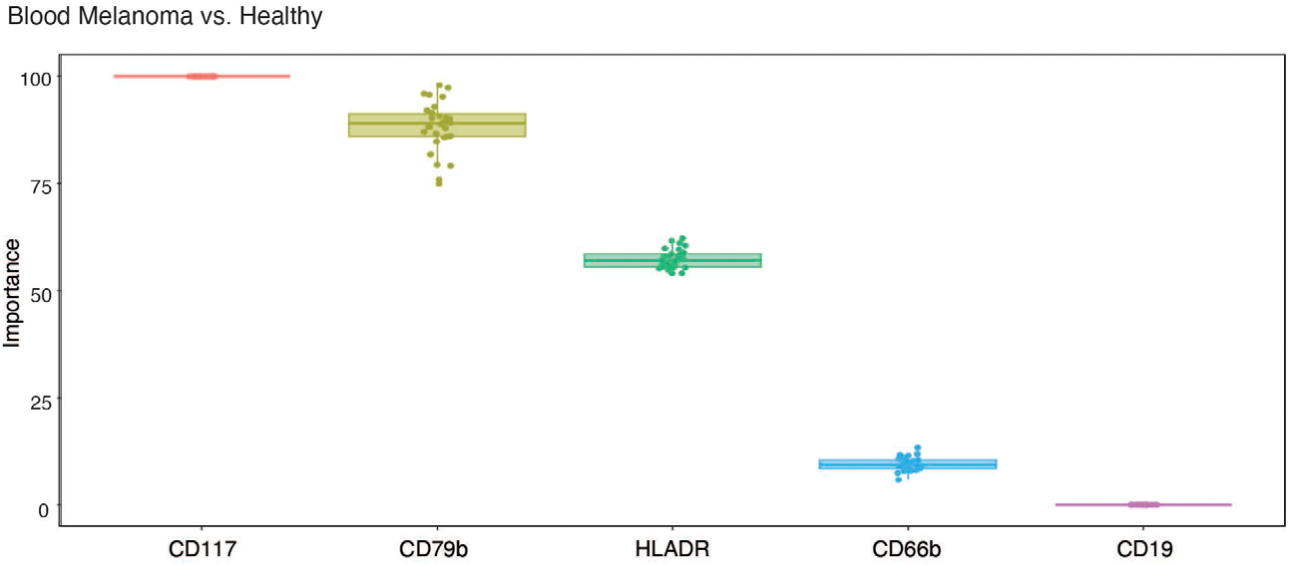
CD79b expression on neutrophils ranks highly in importance for distinguishing melanoma from healthy subjects in blood. Importance of each tested marker among blood neutrophils in distinguishing melanoma subjects from healthy subjects using machine learning.

Because CD79b^+^ neutrophils are elevated in the blood of melanoma patients compared to healthy individuals, we tested their predictive importance using random forest-based machine learning with ten-fold cross-validation. Using the CyTOF data from **Figures 1A, B**, which was generated from melanoma subjects, we examined the importance of CD79b compared to CD117, a marker previously reported to be elevated on neutrophils in the blood of melanoma subjects (38), HLA-DR, a marker that appears on neutrophils in early-stage lung cancer patients (11), and CD19 and CD66b, as controls markers for B cells and neutrophils, respectively. Interestingly, CD79b expression in blood neutrophils showed a high level of importance rank, and a similarly high level of importance rank compared to CD117, in distinguishing melanoma from healthy subjects (**Figure 3**).

### 3.3 CD79b^+^ neutrophils possess neutrophil features

**Figure 4:**
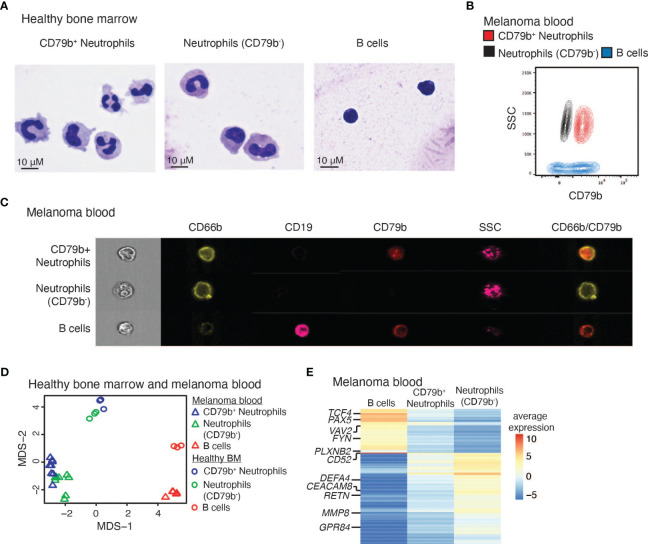
CD79b^+^ neutrophils maintain neutrophil features. **(A)** Cytospins of human bone marrow CD79b^+^ neutrophils (CD3^-^CD56^-^CD19^-^Siglec8^-^CD203c^-^CD86^Lo^CD66b^+^CD79b^+^), CD79b^-^ neutrophils (CD3^-^CD56^-^CD19^-^Siglec8^-^CD203c^-^CD86^Lo^CD66b^+^CD79b^-^), and B cells (CD3^-^CD56^-^CD66b^-^CD19^+^). Representative of n=2 biological replicates per population. The scale bar is 10 μm. **(B)** Flow cytometry of melanoma subject blood CD79b^+^ neutrophils (red), neutrophils (CD79b^-^, black), and B cells (blue) illustrating side scatter (SSC) and CD79b expression, representative of n=41 biological replicates. **(C)** ImageStream analysis of melanoma subject blood CD79b^+^ neutrophils, CD79b^-^ neutrophils, and B cells indicating extracellular protein expression and side scatter (SSC), representative of n=3 biological replicates. **(D)** Bulk RNA-sequencing from CD79b^+^ neutrophils, CD79b^-^ neutrophils, and B cells in human bone marrow and melanoma blood projected in Multi-Dimensional Scaling (MDS) plot. **(E)** Average expression of genes with at least two logFC differences between CD79b^+^ neutrophils vs. B cells and CD79b^+^ neutrophils vs. CD79b^-^ neutrophils by bulk RNA-seq, n=3 biological replicates of each cell type from each tissue.

To further study CD79b^+^ neutrophils, we performed experiments to characterize their appearance and gene expression compared to all remaining CD79b^-^ neutrophils and B cells. Healthy human bone marrow CD79b^+^ neutrophils, CD79b^-^ neutrophils (CD3^-^CD56^-^CD19^-^Siglec8^-^CD203c^-^CD86^Lo^CD66b^+^CD79b^-^), and B cells (CD3^-^CD56^-^CD66b^-^CD19^+^) were sorted and analyzed via cytospin to compare their respective morphologies (**Figure 4A**). CD79b^+^ neutrophils displayed a polymorphonuclear morphology and large cytoplasm, similar to classical neutrophils, though some CD79b^+^ neutrophils presented with a nuclear morphology reminiscent of neutrophils at an earlier developmental stage (**Figure 1B**). With the use of flow cytometry, we also observed a high side-scatter profile amongst the CD79b^+^ neutrophil pool, suggesting a granularity status similar to other neutrophils (**Figure 4B** and **Supplemental Figure 2A**). These data indicate that the CD79b^+^ neutrophils maintain a gross neutrophilic appearance.

To confirm that CD79b^+^ neutrophils represent individual cells and not cellular doublets between neutrophils and B cells, we performed single-cell imaging using ImageStream. Peripheral blood from melanoma subjects was stained for surface B cell and neutrophil markers. We indeed found single cells by brightfield imaging that expressed both CD66b and CD79b, demonstrated a high level of side scatter, and were negative for CD19 (**Figure 4C** and **Supplemental Figure 2B**). Other peripheral blood neutrophils lacked CD79b expression while maintaining CD66b and high side scatter. Conversely, B cells lacked CD66b expression, showed a lower level of side scatter, the gain of CD19, and, sometimes, CD79b expression. Thus, CD79b^+^ neutrophils represent individual single cells that express both B cell and neutrophil markers. Further, we confirmed that CD79b^+^ neutrophils do not express the plasma cell marker CD138 (**Supplemental Figure 2C**) (41). Next, we asked whether CD79b^+^ neutrophils can be generated by co-culturing B cells and neutrophils from melanoma patient blood. At 4 hours and 24 hours of B cell:neutrophil co-culture, we did not observe any additional CD79b^+^ neutrophils beyond those present in the neutrophil-only control culture (**Supplemental Figure 2D**). These data support the notion that the CD79b^+^ neutrophils do not derive from phagocytosis, exosome uptake, trogocytosis, or mergers between neutrophils and B cells.

To more thoroughly define the relationship between CD79b^+^ neutrophils, CD79b^-^ neutrophils, and B cells in healthy human bone marrow and melanoma patient blood, we sorted and profiled these populations by bulk RNA-sequencing. Analysis of global changes in the transcriptomes of these cells by multi-dimensional scaling (MDS) showed that CD79b^+^ neutrophils are more closely grouped with CD79b^-^ neutrophils than B cells in both bone marrow and blood (**Figure 4D**). Unbiased hierarchical clustering of the top 10% variable genes across all samples confirmed this observation (**Supplementary Figure 3** and **Supplementary Table 2**). The CD79b^+^ neutrophil compartment maintained the expression of conventional neutrophil markers such as *S100A8/9* and *CSF3R*, immaturity markers such as *MMP8/9*, and transcription factors *CEBPE* and *CEBPA.* CD79b^+^ neutrophils also lacked expression of some typical B cell genes such as *CD19* and *MS4A1* (encoding CD20) (**Supplementary Figure 3A** and **Supplementary Table 2**). To detail how CD79b^+^ neutrophils differ from B cells and neutrophils, we identified 314 DEGs (cutoff of ≥2 log fold change) between either CD79b^+^ neutrophils and B cells or between CD79b^+^ neutrophils and CD79b^-^ neutrophils (**Figure 4E** and **Supplementary Table 3**). Notably, CD79b^+^ neutrophils had a higher expression of B cell-associated markers, including *PAX5, FYN, TCF4*, and *VAV2*, than CD79b^-^ neutrophils. These transcriptional profiles show that the expression of these genes in CD79b^+^ neutrophils is intermediate between B cells and neutrophils.

### 3.4 CD79b^+^ neutrophils have an increased propensity for NETosis

**Figure 5:**
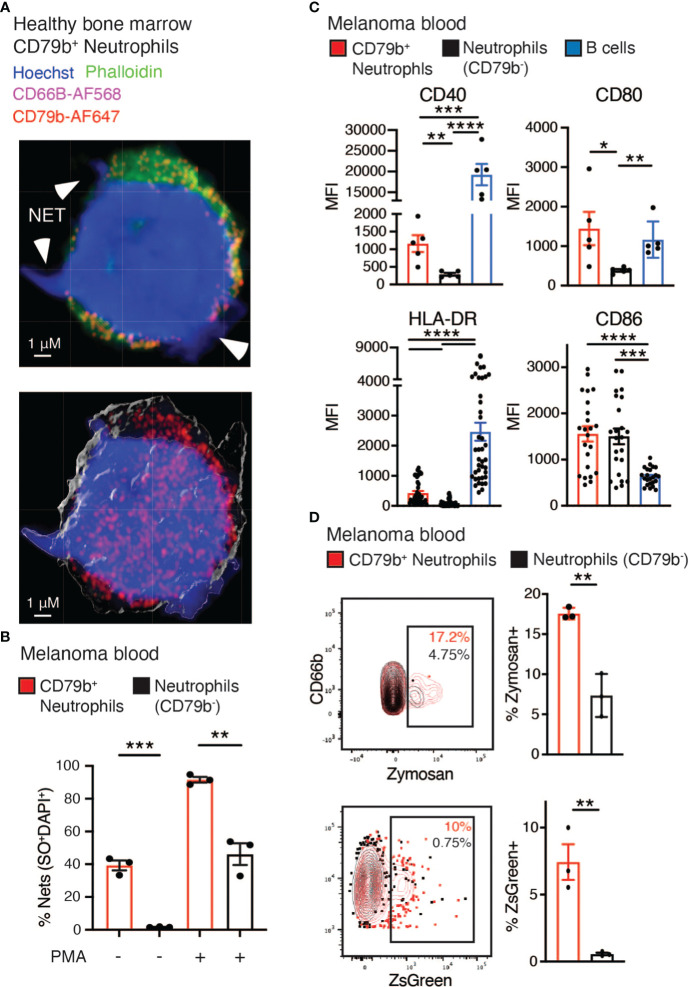
Increased phagocytosis and co-stimulatory molecule expression found among CD79b^+^ neutrophils. **(A)** Representative confocal image of healthy human bone marrow CD79b^+^ neutrophils (CD3^-^CD56^-^CD19^-^Siglec8^-^CD203c^-^CD86^Lo^CD66b^+^CD79b^+^). CD79b is displayed in red, CD66b is displayed in pink, and arrowheads indicate NETs illustrated by protruding DNA stained with Hoechst (blue), n=3 technical replicates for each of n=2 biological replicates. The scale bar is 1 μm. **(B)** The measure of NETosis by flow cytometry in CD79b^+^ neutrophils (red) and CD79b^-^ neutrophils (black, CD3^-^CD56^-^CD19^-^Siglec8^-^CD203c^-^CD86^Lo^CD66b^+^CD79b^-^) from melanoma subject blood. Whole blood was cultured +/- phorbol 12-myristate 13-acetate (PMA) for 3 hours. NETs indicated by extracellular Sytox Orange (SO) and DAPI staining, n=3 per condition, representative of 2 biological replicates. **(C)** Mean fluorescence intensity (MFI) of antigen-presenting machinery in CD79b^+^ neutrophils, CD79b^-^ neutrophils, and B cells (CD3^-^CD56^-^CD66b^-^CD19^+^) from human melanoma subject blood by flow cytometry. n=6 for CD40 and CD80, n=40 for HLA-DR and CD86. **(D)** Whole blood from melanoma subjects was co-cultured with zymosan (top panel) or ZsGreen^+^ B16-F10 tumor cells (bottom panel) for 2 hours. Zymosan or ZsGreen^+^ tumor cell uptake was measured by flow cytometry in CD79b^+^ neutrophils and CD79b^-^ neutrophils, n=3 per condition, representative of 2 biological replicates. * p< 0.05, ** p<0.01, *** p<0.001, **** p<0.0001 by two-sided, unpaired t-test for single comparisons and RM one-way ANOVA, with the Geisser-Greenhouse correction with Tukey’s *post hoc* test for multiple comparisons with paired values panel **(C)**.

We next measured several neutrophil-specific functions in CD79b^+^ neutrophils compared to other blood neutrophils in subjects with melanoma. NETosis is a unique neutrophilic function that has recently been implicated in promoting tumor development and metastasis (42). Via fluorescent confocal imaging, we were able to observe CD79b^+^ neutrophils expressing CD79b (red) and producing NETs, as indicated by the extrusion of DNA (blue) into the extracellular space (**Figure 5A**). We assessed NETosis in the CD79b^+^ neutrophil pool by measuring the co-staining of SyTOX Orange and DAPI to detect extracellular DNA appended to cells by flow cytometry. We found that CD79b^+^ neutrophils generate NETs at greater rates than other neutrophils, at baseline and after stimulation with phorbol 12-myristate 13-acetate (PMA) (**Figure 5B**) (20). NETosis was also measured in healthy human bone marrow. Our results again showed that bone marrow CD79b^+^ neutrophils produce NETs at a greater rate than CD79b^-^ neutrophils, similar to what we observed in the blood (**Supplemental Figure 4A**). We further found that CD79b^+^ neutrophils produced ROS and superoxide at levels similar to those of CD79b^-^ neutrophils (**Supplemental Figure 4B**). These data suggest that CD79b^+^ neutrophils maintain many canonical neutrophilic functions, but they possess an improved capacity for NETosis, especially without PMA stimulation, compared to other neutrophils.

### 3.5 Increased phagocytosis and co-stimulatory molecule expression found among CD79b^+^ neutrophils

Next we asked if CD79b^+^ neutrophils display features typically associated with B cells. We first assessed antibody production by CD79b^+^ neutrophils. These cells did not express either IgM or IgD on their cell surface, suggesting that they do not produce antibodies or express fully-assembled B cell receptors (**Supplemental Figure 4C**) (43). Antigen presentation is another B cell function often absent from neutrophils, and indications that CD79b^+^ neutrophils express antigen presentation-related machinery were present in the CyTOF analysis (**Figure 1B**). Using flow cytometry, we found that CD79b^+^ neutrophils expressed higher levels of the antigen presentation-related proteins, namely CD40, CD80, and HLA-DR, compared to other neutrophils in melanoma subjects (**Figure 5C**). However, CD79b^+^ neutrophils did not express higher levels of CD86 by flow cytometry (**Figure 5C**). This expression profile mirrors the expression pattern observed in the B cells from the same subjects. Further, a closer look at the cytospin images in **Figure 4A** reveals CD79b^+^ neutrophils possessed dendrites resembling those of dendritic cells. This observation is consistent with their increased antigen presentation-related cell surface protein expression.

Given that the CD79b^+^ neutrophil subset expressed antigen presentation markers, we assessed the ability of these cells to phagocytose. Whole blood from melanoma subjects was co-cultured with either Zymosan or B16-F10 tumor cells labeled with ZsGreen, a lysosomal stable fluorescent protein (44, 45), for 2 hours and then analyzed by flow cytometry. CD79b^+^ neutrophils took up a greater level of Zymosan and ZsGreen+ tumor cells than CD79b^-^ neutrophils obtained from the same melanoma subject blood sample (**Figure 5D**). Because phagocytosed antigens could be used for antigen presentation, these support a role for CD79b^+^ neutrophils as antigen presenters, which will need to be further explored.

## 4 Discussion

Here we described the novel expression of CD79b among human neutrophils and show that these cells are restricted to the bone marrow during steady-state but appear in the periphery during cancer. We found that CD79b^+^ neutrophils were elevated in the blood of melanoma patients with early-stage disease rather than late-stage disease. Using machine learning, we found that CD79b expression among neutrophils in the blood ranks highly in importance for distinguishing healthy from melanoma blood. CD79b expression in neutrophils is concordant with a capacity for these cells to undergo NETosis in unstimulated conditions, superior phagocytosis, and expression of antigen-presenting machinery. CD79b expression in neutrophils could act as a disease biomarker and could indicate a certain functional state among neutrophils.

There are only a few reports of immune cells with overlapping neutrophil and B cell phenotypes (35, 46–48). High-dimensional studies have shown the presence of B cell-related gene expression in neutrophils (35, 47). Through trajectory analysis, Wilk et al. defined a population of ‘developing neutrophils’ in subjects with COVID-19 that seemed to derive from plasmablasts and overlap in marker expression with immature neutrophils (i.e., *MPO, ELANE*) *(* 47). Experiments *in vitro* have found that overexpression of CEBPβ or CEBPε in immortalized primary B cells produces granulocytes (46). Further, in hematopoietic tumors, Pax5 seemed to regulate the myeloid versus B cell lineage phenotypes in the cancer cells (48). The spontaneous downregulation of Pax5 in cancer cells led to a myeloid phenotype, while enforced Pax5 expression produced a lymphoid-like tumor. We observed that Pax5 is one of the transcription factors expressed in CD79b^+^ neutrophils (**Figure 4E**). Our finding is unique because CD79b expression is largely thought to be restricted to the B cell lineage and has not previously been shown to be biologically meaningful among neutrophils. Together our study and previous work suggest that the neutrophil and B cell programs are not as segregated as traditional views of hematopoiesis endorse.

CD79b^+^ neutrophils may be sharing selected gene expression with B cells to drive unusual phenotypes in neutrophils. Though neutrophils are not specialized in antigen presentation, they have been previously reported to present antigens during disease (11–13, 49–51). For example, antigen-presenting neutrophils are described in tumoral tissue from early-stage lung cancer and have been reported to have a role in stimulating T cells in the lymph node in non-metastatic head and neck cancer (11, 13). The CD79b^+^ neutrophil phenotype aligns with these antigen-presenting neutrophils, as CD79b^+^ neutrophils are also present in the blood of melanoma patients at the early stages of disease. Another report has described a population of neutrophils able to stimulate memory CD4^+^ T cells (49). Unlike other studies, our work begins to connect the neutrophil antigen presentation machinery expression to proteins typically expressed in B cells. Further work is needed to confirm antigen presentation function among CD79b^+^ neutrophils, to understand how the B cell program regulates antigen presentation phenotypes in neutrophils, and whether this function in CD79b^+^ neutrophils impacts tumor progression.

Because neutrophils have a high turnover rate and are the most abundant cell type in the blood (7, 16), they may act as indicators of disease. Specific subsets of neutrophils are elevated in cancer patients’ blood, particularly those found in the bone marrow at a steady state (9, 17). We show that CD79b^+^ neutrophils are specifically elevated in the blood of subjects with early-stage melanoma. We hypothesize that CD79b expression among neutrophils could be an early biomarker of melanoma incidence. The expression of other unique markers among neutrophils should be explored in the blood during disease to identify additional potential blood-based indicators of illness.

This study was plagued by one major limitation: the age of whole blood samples from melanoma patients. Many samples analyzed and used to interrogate further CD79b^+^ neutrophils were shipped overnight on cold-pack. We acknowledge this is not ideal. As CD79b^+^ neutrophils were still rare among blood cells from melanoma patients, sourcing samples from a cooperative biobank was critical to obtain the number of samples and cells needed for this study. Additionally, the major finding, the presence of CD79b^+^ neutrophils in melanoma patient blood, was repeated in the cohort age-match cohort sourced from the University of Southampton, where healthy donor and matched melanoma patient samples were processed within hours of the blood draw (**Figure 2D**). This correlation in findings between fresh and shipped samples is concordant with Johnson et al., who found immunophenotyping 24 hours after the draw was consistent with a fresh sample, even among neutrophils (52).

In summary, our study defines CD79b expression among neutrophils as correlative with early-stage melanoma incidence. The current work, however, does yet fully define the function or elucidate the pro- versus anti-tumor properties of CD79b^+^ neutrophils in melanoma. Further work in mouse models and exploration in larger datasets with relevant outcome data is needed to define CD79b^+^ neutrophils as a functional subset in melanoma fully. Regardless of results related to function, the current data suggest CD79b^+^ neutrophils enrichment in early-stage melanoma patients makes them poised to be developed further as a biomarker of disease. Further work will define the CD79b^+^ neutrophil’s role in cancer progression and determine its usefulness as a biomarker and therapeutic target.

## Data availability statement

The datasets presented in this study can be found in online repositories. The names of the repository/repositories and accession number(s) can be found below: https://www.ncbi.nlm.nih.gov/geo/, GSE154777.

## Ethics statement

The studies involving humans were approved by Institutional Review Board of La Jolla Institute for Immunology and in accordance with US Department of Health and Human Services Policy for the Protection of Human Research Subjects; Southampton and Southwest Hampshire Research Ethics Committee. The studies were conducted in accordance with the local legislation and institutional requirements. The participants provided their written informed consent to participate in this study.

## Author contributions

MM, HD, and CH conceptualized the project. MM, NG, YZ, EH, SL, GS, WK, SC, and PV performed experiments. MM, HD, AA, NC, WK, and PV performed data analysis. CO provided human samples. MM, HD, DA, and CH composed the manuscript. All authors reviewed and edited the manuscript. MM and HD are co-first authors. MM is listed first for completing this project after HD made the initial discovery. All authors contributed to the article and approved the submitted version.
