# Supplementary material for: Human CD79b+ neutrophils in the blood are associated with early-stage melanoma
Source: Front Immunol. 2023 Oct 31;14:1224045. doi: 10.3389/fimmu.2023.1224045 (PMC10643866; doi:10.3389/fimmu.2023.1224045)
Supplement: Supplementary file 1 [file DataSheet_1.docx]

Supplementary Material

Human CD79b^+^ neutrophils in the blood are associated with early-stage melanoma.

**Melissa A. Meyer^1,†^, Huy Q. Dinh ^1,2†^, Ahmad Alimadadi^1^, Daniel J. Araujo^1^, Nandini Chatterjee^1^, Norma A. Gutierrez^1^, Yanfang P. Zhu^1,3^, Emma L. Hunter^1^, Shu Liang^1^, Gregory Seumois^1^, William B. Kiosses^4^, Sergio D. Catz^5^, Pandurangan Vijayanand^1^, Christian Ottensmeier^1,6^, Catherine C. Hedrick^1, 7^***

^†^These authors contributed equally to this work.

*** Correspondence:** Catherine C. Hedrick: LHEDRICK@augusta.edu

## Supplementary Figures


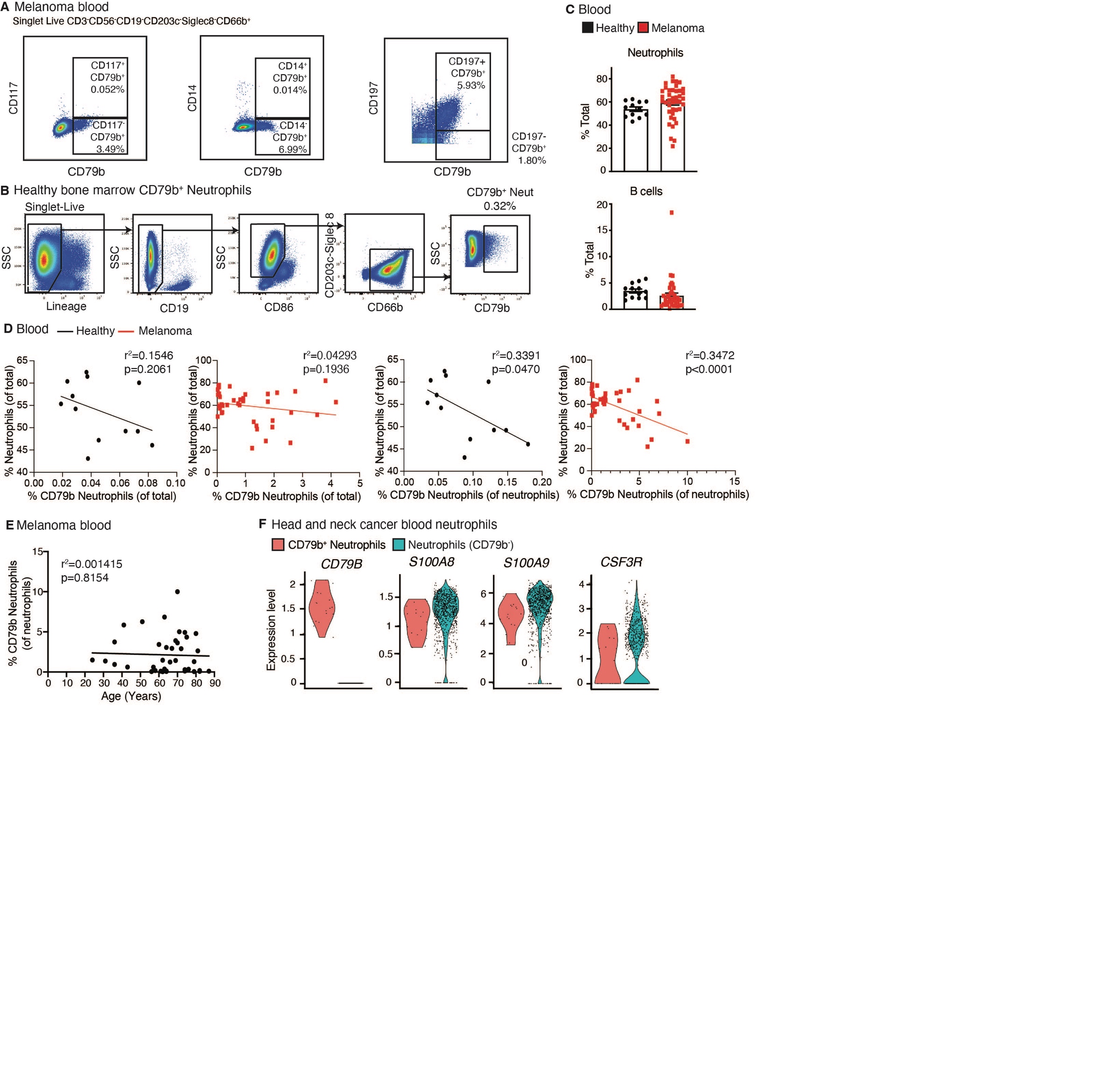


**Supplementary Figure 1.** (A) Flow cytometry of melanoma patient blood neutrophils (CD3^-^CD56^-^CD19^-^Siglec8^-^CD203c^-^CD66b^+^) illustrating CD117 versus CD79b expression and CD14 versus CD79b expression. CyTOF of melanoma patient blood neutrophils illustrating CD197 versus CD197 expression. Flow cytometry representative of n=41. CyTOF representative of n=17. (B) Flow cytometry gating strategy for CD79b^+^ neutrophils in healthy human bone marrow. (C) Frequency of neutrophils (CD3^-^CD56^-^CD19^-^CD203c^-^Siglec8^-^CD66b^+^) and B cells (CD3^-^CD56^-^CD66b^-^CD19^+^) in healthy human control and human melanoma patient blood by flow cytometry, n=12 healthy controls, n=41 melanoma patients. (D) Correlation between CD79b^+^ neutrophil frequency of total cells and neutrophils and neutrophil frequency of total cells in healthy human control and human melanoma patient blood. (E) Correlation between human melanoma patient blood CD79b^+^ neutrophil frequency by flow cytometry and patient age, n=35. (F) Violin plots show the identification of CD79b^+^ neutrophils (*CD79B*+) among head and neck cancer peripheral blood neutrophils in scRNA-seq data. Peripheral blood neutrophils identified by high expression of *CSF3R* (5.85% of peripheral blood mononuclear cells). CD79b^+^ neutrophils (2% of neutrophils) exclusively expressed *CD79B* while retaining high expression of neutrophil markers (*S100A8/9* and *CSF3R*.


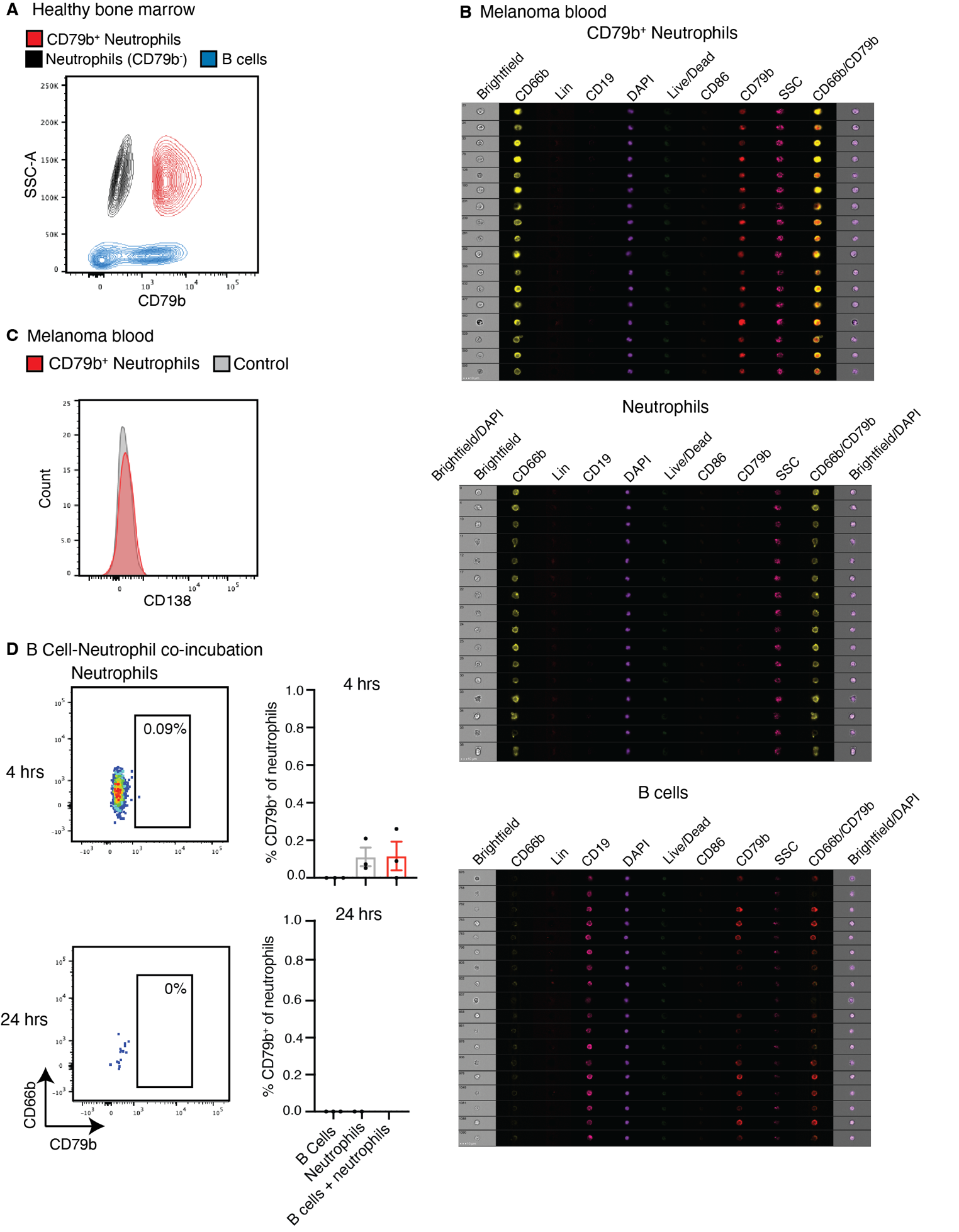


**Supplementary Figure 2.** (A) Flow cytometry of healthy human bone marrow CD79b^+^ neutrophils (red), neutrophils (CD79b^-^, black), and B cells (blue) illustrating side scatter (SSC) and CD79b expression. Representative of n=8 biological replicates. (B) ImageStream analysis of melanoma patient blood CD79b^+^ neutrophils (CD3^-^CD56^-^CD19^-^CD203c^-^Siglec8^-^CD66b^+^CD79b^+^), neutrophils (CD3^-^CD56^-^CD19^-^CD203c^-^Siglec8^-^CD66b^+^CD79b^-^), and B cells (CD3^-^CD56^-^CD66b^-^CD19^+^) indicating extracellular protein expression and side scatter (SSC). Cellular replicates of each cell type are shown. Representative of n=3 biological replicates. (C) CD138 expression measured by flow cytometry on CD79b^+^ neutrophils (CD3^-^CD56^-^CD19^-^CD203c^-^Siglec8^-^CD66b^+^CD79b^+^) from melanoma patient blood. Representative of n=3 biological replicates. (D) Neutrophils (CD3^-^CD56^-^CD19^-^CD203c^-^Siglec8^-^CD66b^+^) and B cells (CD3^-^CD56^-^CD66b^-^CD19^+^) were cultured individually and co-cultured for 4 or 24 hours. Culture products were analyzed by flow cytometry for neutrophils (CD3^-^CD56^-^CD19^-^CD203c^-^Siglec8^-^CD66b^+^) and neutrophil CD79b expression was assessed, n=3 biological replicates.


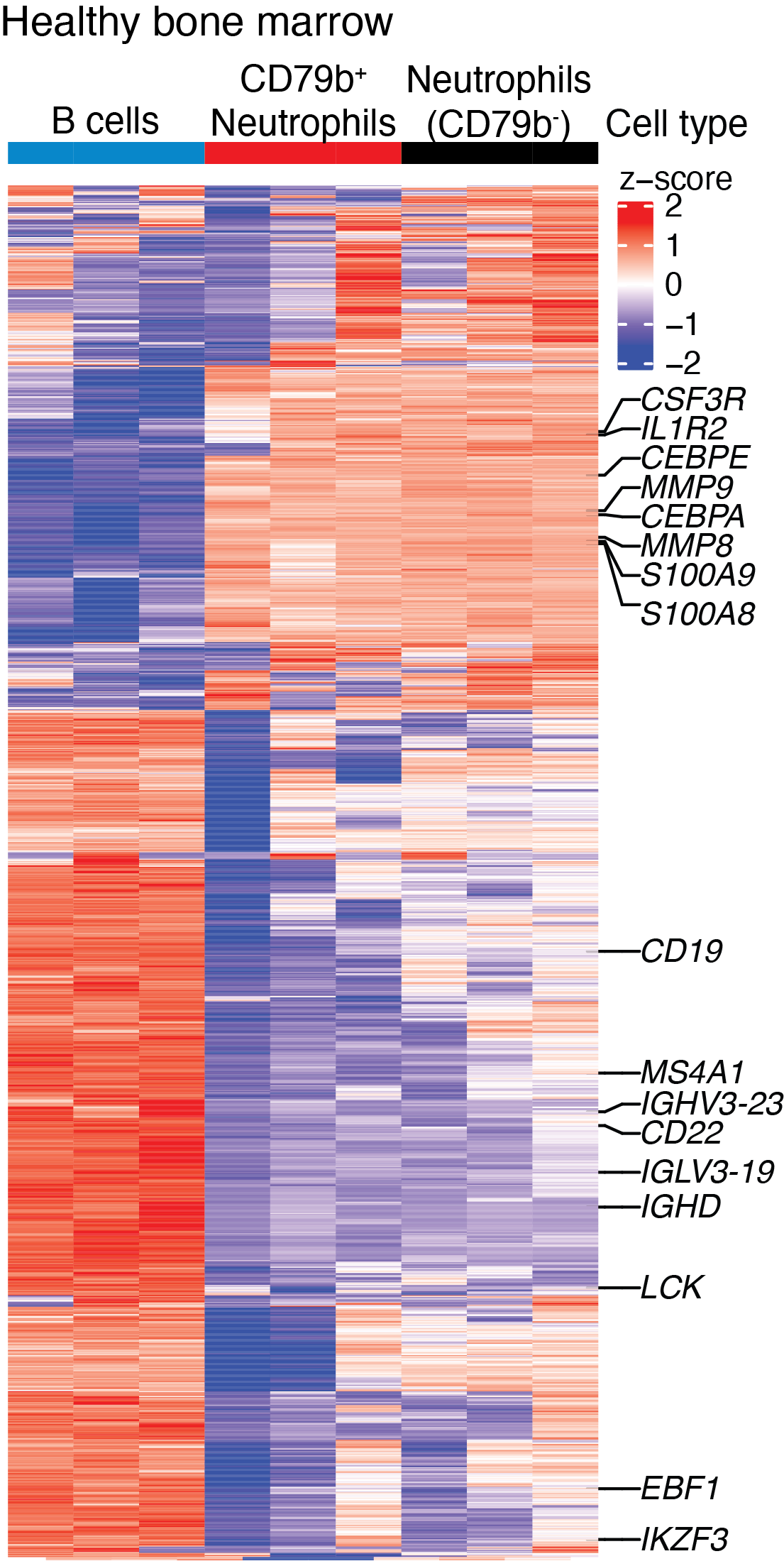


**Supplementary Figure 3.** Heatmap of top 10% variable expressed genes (ordered by standard deviation) across nine samples from Fig. 2C. Highlighted in texts are representative neutrophil and B cell genes.


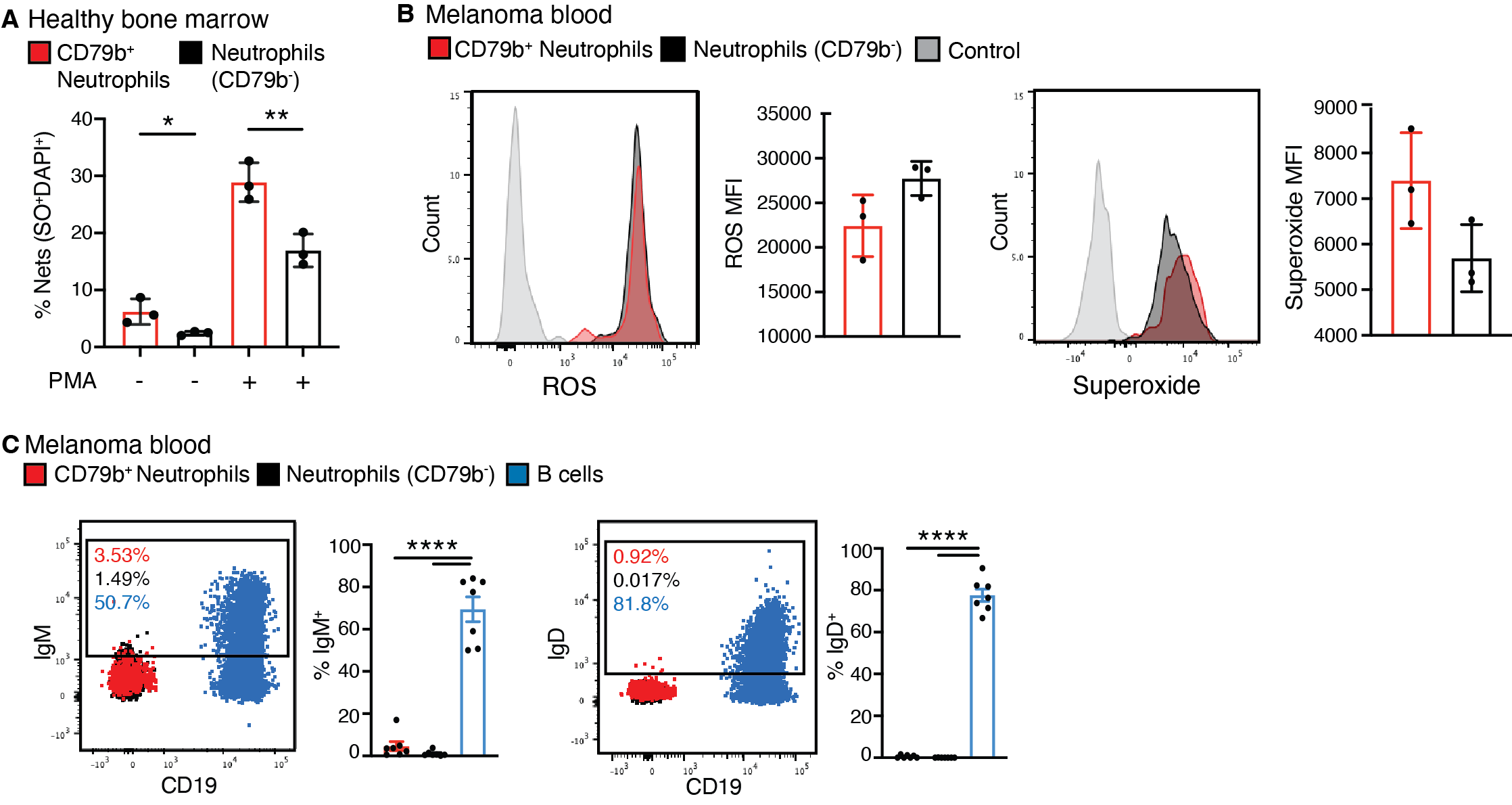


**Supplementary Figure 4.** (A) The measure of NETosis by flow cytometry in CD79b^+^ neutrophils (red) and neutrophils (CD79b^-^, black) from healthy human bone marrow. Whole bone marrow was cultured +/- phorbol 12-myristate 13-acetate (PMA) for 3 hours. NETs indicated by extracellular Sytox Orange (SO) and DAPI staining, n=3 per condition, representative of 3 biological replicates. (B) ROS and superoxide were measured by flow cytometry using the Abcam ROS/Superoxide Detection Assay Kit in human melanoma patient blood CD79b^+^ neutrophils (CD3^-^CD56^-^CD19^-^CD203c^-^Siglec8^-^CD86^Lo^CD66b^+^CD79b^+^) and neutrophils (CD3^-^CD56^-^CD19^-^CD203c^-^Siglec8^-^CD86^Lo^CD66b^+^CD79b^-^), n=3. (C) Cell surface expression of IgM and IgD by flow cytometry in CD79b+ neutrophils (red), neutrophils (CD79b^-^, black), and B cells (blue). n=7 biological replicates. * p< 0.05, ** p<0.01, and **** p<0.0001 by two-sided, unpaired t-test for single comparisons and by one-way ANOVA with Tukey’s post hoc test for multiple comparisons.
